# Self-organizing complex networks with AI-driven adaptive nodes for optimized connectivity and energy efficiency

**DOI:** 10.1038/s41598-025-28035-0

**Published:** 2025-12-24

**Authors:** Azra Seyyedi, Mahdi Bohlouli, SeyedEhsan Nedaaee Oskoee

**Affiliations:** 1 Department of Physics, Institute for Advanced Studies in Basic Sciences (IASBS), Zanjan, 45137-66731 Iran; 2 Research and Innovation Department, Petanux GmbH, Bonn, 53175 North Rhine-Westphalia Germany

**Keywords:** Applied physics, Complex networks

## Abstract

High connectivity and robustness are essential in distributed networks, ensuring resilience, efficient communication, and adaptability. Optimizing energy consumption is also crucial for sustaining energy-constrained networks and extending their operational lifespan. In this study, we introduce an Artificial Intelligence (AI)-enhanced self-organizing network model, where each adaptive node autonomously adjusts its transmission range to optimize network connectivity while lowering energy consumption. Building on our previous Hamiltonian-based methodology, which is designed to achieve globally optimized states of complete connectivity with minimal energy usage, this research integrates a Multi-Layer Perceptron (MLP)-based decision-making model at each node. By leveraging a dataset from the Hamiltonian approach, nodes independently learn and adapt transmission range based on local conditions, leading to emergent global behaviors characterized by high connectivity and resilience to structural disruptions. This distributed, AI-driven adaptability allows nodes to make context-aware range adjustments autonomously, enabling the network to maintain its optimized state over time. Simulation results show that AI-driven adaptive nodes achieve stable connectivity, robustness, and energy efficiency across different conditions, including static and mobile scenarios. This work contributes to the growing field of self-organizing networks by demonstrating AI’s potential to enhance complex network design, fostering scalable, resilient, and energy-efficient distributed systems.

## Introduction

In distributed networks, achieving high connectivity and robustness is essential for ensuring resilience, efficient communication, and adaptability in dynamic environments. Robustness ensures that the network remains functional despite node failures, while resilience enables it to adapt and recover from disruptions^1,2^. Distributed networks, where each node operates independently and without centralized control, play a crucial role in wireless sensor networks (WSNs)^3^, emergency response systems^4^, Internet of Things (IoT) ecosystems^5^, and autonomous systems^6^. However, energy consumption remains a major concern, as many of these networks consist of energy-constrained devices with limited battery life^7^. Thus, achieving efficient energy usage while maintaining network performance is vital for ensuring the longevity and sustainability of such systems.

Traditional approaches for ensuring connectivity and robustness often rely on static configurations or centralized control mechanisms, which may not be suitable for large-scale, distributed, or mobile networks. In contrast, self-organizing networks allow nodes to autonomously establish and maintain connectivity through local interactions, particularly when combined with advanced decision-making algorithms. Early research on self-organizing networks focused on local interactions. Barabási and Albert^8^ introduced scale-free networks, demonstrating how preferential attachment enables resilient structures that maintain connectivity despite node failures. Later studies^9^ explored local decision-making for achieving global resilience, particularly in mobile ad-hoc networks (MANETs)^10^ and WSNs^11^, where optimizing communication protocols has been crucial for improving network efficiency and longevity^12^. To enhance network adaptability, adaptive control mechanisms have been proposed, allowing nodes to dynamically adjust their transmission power based on local topology and energy constraints^13^.

With the rise of machine learning (ML) and AI, intelligent adaptation has become a promising avenue for improving self-organizing networks. In self-organizing, infrastructure-free systems, AI becomes practically useful when inference is executed in a fully distributed manner. While training can be conducted offline or in hybrid modes, true autonomy depends on distributed deployment at runtime^14^. The literature on AI/ML shows a clear shift from centralized, controller-based methods toward decentralized and fully self-organized approaches, and this evolution changes which methods are practical and which research problems dominate^15,16^. Supervised learning, long the workhorse for tasks such as traffic prediction^17^, link-quality estimation^18^, and fault detection^19^, remains predominantly a centralized technique because local nodes typically lack sufficient labeled data, although federated and distributed training schemes are an important and growing exception^20^.

Unsupervised and self-supervised methods are powerful in principle for local feature learning and anomaly detection without labels^21^, but they are still under-developed for fully distributed deployments. Reinforcement learning (RL) spans both worlds^22^. Early RL applications assumed a central view, like Software-Defined Networking (SDN) routing^23^, power allocation^24^, Unmanned Aerial Vehicle (UAV) trajectory planning^25^, while recent work, specifically Multi-agent RL (MARL) increasingly targets decentralized scenarios including distributed routing, medium access, energy balancing, where agents learn from local reward signals^26–28^. Graph neural networks (GNNs) conceptually map well to self-organizing systems because of their localized message-passing semantics, yet most GNN research so far trains models centrally; only nascent efforts toward true message-passing or locally executed GNNs aim to close that gap^29,30^. Hybrid approaches (GNN+RL, GNN+supervised) are already successful in centralized settings and represent a promising, if technically challenging, frontier when adapted to distributed settings^31,32^.

Despite extensive work on connectivity, most studies either (i) measure or predict connectivity rather than actively optimizing it, or (ii) pursue connectivity as a secondary objective while prioritizing other metrics (energy, throughput) or making modeling assumptions incompatible with a fully distributed setting. Representative classes of prior work include motion/mobility-control that restore connectivity through planned robot/UAV/sensor trajectories^33–37^; placement/relay/deployment solved by ILP or centralized heuristics^38–44^; population-based optimizers that add links or choose placements but are centralized and computationally costly^38,42,44–47^; topology/power/algebraic approaches that assume staged or centralized procedures or optimize different objectives^48–55^; and routing-layer strategies that preserve connectivity through protocol design rather than by changing physical/topological relationships^56^.

In contrast, our work formulates and studies a fully distributed framework: identical nodes, no manager, and strictly local observations/actions. Local policies induce spatial and topological adaptations that collectively maximize global connectivity under energy constraints. This framing is intentionally agnostic to platform-specific details and targets the challenging regime of random deployment, random motion, and low node density – a regime underrepresented in prior, centrally guided or application-specific studies.

In our previous work^57^, we introduced a Hamiltonian-based methodology that allowed nodes to autonomously adjust their transmission power and form links based on their spatial proximity and transmission range. This approach guided the network toward a globally optimized state, achieving near-complete connectivity, high robustness, and minimal energy consumption. The Hamiltonian framework demonstrated how self-organizing topologies could sustain optimized connectivity even in the presence of local or major disruptions. While being effective, this approach relied on a predefined, numerical method, leaving potential for further adaptability and resilience through learning-based techniques.

In this study, we introduce an AI enhanced methodology that leverages deep learning to enhance node adaptability within the self-organizing complex network. Specifically, each node is equipped with two Multi-Layer Perceptron (MLP) models trained on datasets generated from our Hamiltonian-based approach. These models enable each node to autonomously learn optimal transmission power adjustments and link formation strategies based on local conditions. By allowing nodes to make context-aware decisions, this approach dynamically optimizes connectivity, robustness, and energy efficiency in a fully distributed manner. Indeed, by decentralizing decision-making, we enable network optimization to emerge from coordinated, AI-driven actions of individual nodes. This enhanced adaptability allows the network to dynamically respond to environmental changes while maintaining optimal performance over time.

This Hamiltonian-to-ML distillation pipeline aggregates physics-grounded labels offline to train a compact supervised policy (two ultra-lightweight MLPs) identically deployed on every node. This removes the need for costly online optimization or unsafe exploration while preserving the global trade-off between connectivity and energy. Methodologically, the pipeline fills a gap between heavy, structure-aware models and fragile heuristics: unlike GNN surrogates, which require graph-level information and richer runtime message exchange, and unlike MARL, which demands prolonged online interaction and extensive training, our approach produces deterministic, sample-efficient policies with minimal per-node computation and communication. Relative to federated schemes and ad-hoc heuristics, it simplifies training while maintaining principled interpretability. The method intentionally trades some representational expressivity for clear deployability, low runtime cost, and interpretability, which are the properties that enable practical use on battery-limited, infrastructure-free networks, and which are empirically validated by full connectivity across our test scenarios.

Furthermore, simulation results illustrate how AI-driven adaptive nodes, functioning in a self-organizing complex network, achieve superior robustness, resilience, and energy efficiency in various scenarios compared to conventional approaches. This study demonstrates the effectiveness of this approach in both static and mobile network environments, demonstrating its potential for real-world applications. It highlights the benefits of integrating deep learning within complex network design, paving the way for the development of scalable, resilient, and energy-efficient distributed systems across diverse applications.

## Results

**Fig. 1:**
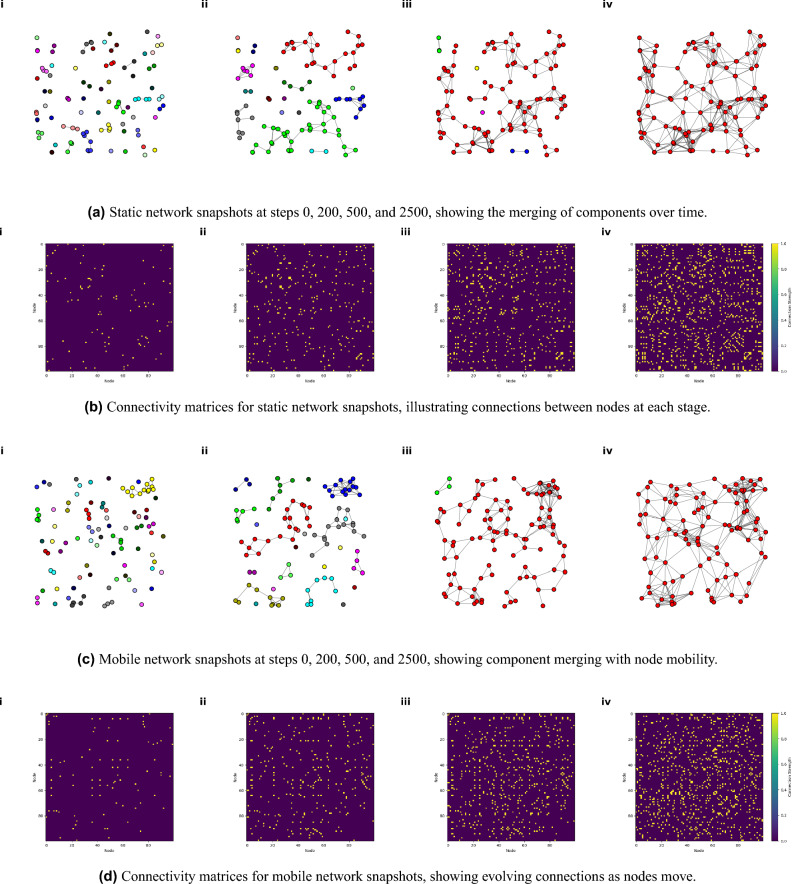
Snapshots and connectivity matrices of the complex network of adaptive nodes at steps 0, 200, 500 and 2500. (**a**) Static network snapshots showing the evolution of node connectivity and the merging of components over time until reaching full connectivity, with nodes displayed in different colors to distinguish components. (**b**) Connectivity matrices for static network snapshots, where yellow cells indicate connections between node pairs. (**c**) Mobile network snapshots, similar to (**a**), showing node connectivity with movement introduced over time. (**d**) Connectivity matrices for mobile network snapshots, reflecting network progression with node movement. The networks are spatial, with x and y coordinates considered in the snapshots (**a**) and (**c**). Connectivity matrices (**b**) and (**d**) are symmetric, reflecting undirected, bidirectional links.

This section begins by analyzing the network’s performance in terms of the evaluation metrics under varying conditions. At first, some snapshots of dynamic behavior and connectivity progression in our adaptive networks with both static and mobile nodes over time are illustrated in Fig. 1. Subfigures 1a and b display evolution of adaptive network with static nodes and subfigures 1c and d offer an analogous progression for the mobile adaptive network. Subfigures 1a and c show snapshots of the networks at different steps related to various stages. In each snapshot, nodes are represented based on their spatial coordination. They display node connectivity at different steps, with colors distinguishing separate components to highlight merging behavior as the network evolves. Each snapshot shows that, initially, multiple components are present, which gradually merging into a single connected component, achieving full connectivity. By step 2500, all nodes belong to a single component (indicated by uniform color), confirming full connectivity with fewer links than a fully saturated network, which highlights an efficient, adaptive structure.

Figure 1b and d show the corresponding connectivity matrices for each step, with yellow cells representing connected node pairs. These matrices provide an additional view into the network’s structural evolution, where increasing yellow areas reflect the growing interconnectivity of nodes. The initial sparsity of yellow cells at step 0 indicates limited connections, while the dense yellow region at step 2500 reflects a cohesive, fully connected network. Symmetry in the matrices reflecting the undirected, bidirectional nature of the connections. In Fig. 1c , It seems that the mobility of nodes facilitates faster component merging. Figure 1d reinforces this observation: node mobility leads to a faster increase in connectivity over time, as evidenced by the earlier appearance of dense yellow regions than in the static network.

These visualizations underscore the adaptive and efficient connectivity mechanisms in both static and mobile networks. This efficiency is key in adaptive networks, as it demonstrates that both types of networks achieve maximum connectivity with low link redundancy. This characteristic is beneficial for reducing overall network energy usage, while ensuring that all nodes remain connected under dynamic conditions.

**Fig. 2:**
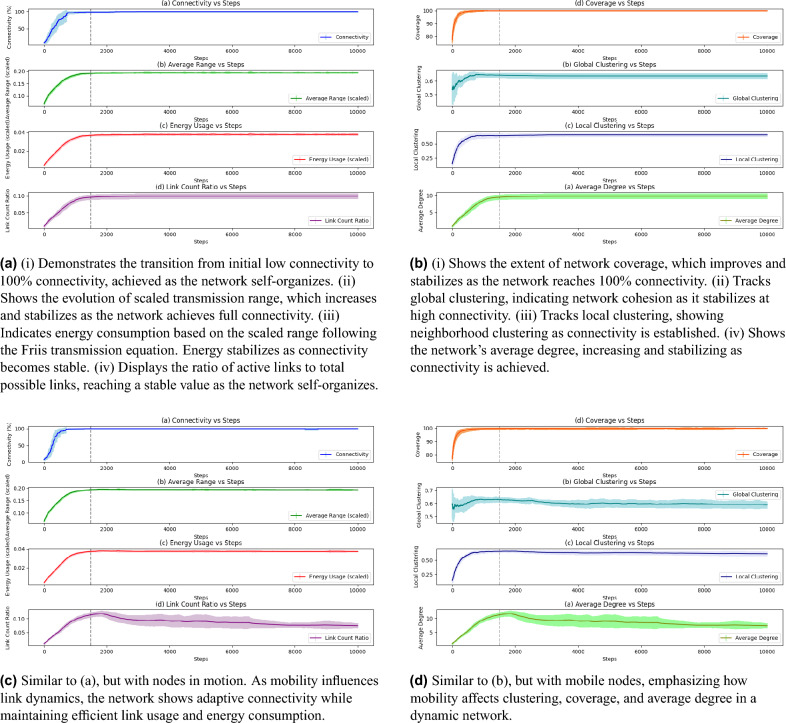
This figure presents the evolution of key network metrics over 10,000 steps, averaged over 10 different initial configurations for each network type. The error bars reflect the variability among these simulations, capturing the robustness of the results despite differences in initial node configurations. Subfigures (**a**) and (**b**) depict metrics for the complex network of static adaptive nodes, while (**c**) and (**d**) show equivalent metrics for mobile adaptive nodes, all at a node distribution density of 0.05. The metrics tracked include connectivity, average range, energy usage, link count ratio, coverage, global clustering, local clustering, and average degree. The data reveals how both static and mobile networks reach stable connectivity and other desired states, with mobile networks adapting under the challenge of random movement dynamics.

In the following, Fig. 2, we present an in-depth analysis of dynamics of the adaptive complex networks with both static and mobile nodes over a long simulation time (over 10,000 steps), providing insight into network behaviors at different stages. The evaluation is conducted using key performance metrics and supporting indicators, providing detailed insights into the network’s behavior over time. Each metric is analyzed and and discussed comprehensively to highlight the network’s performance. In each experiment, the network’s behavior was averaged across 10 simulations with identical parameters but different initial configurations to ensure statistical reliability. This averaging approach ensures that the results are not biased by any single starting configuration, providing a more comprehensive picture of network behavior. The error bars in each plot indicate the variability among these initial configurations, showcasing the consistency and reliability of the connectivity patterns and other metrics achieved by the adaptive network algorithm.

Each metric highlights different aspects of the network’s performance. Notable points are included in the following. Connectivity Progression: In both static Fig. 2a and mobile Fig. 2c networks, initial connectivity starts low and progressively, reaches 100% around step 1500. Beyond this point, the connectivity stabilizes and maintains a consistent value, showing successful network adaptation to full connectivity in a short time and its ability to sustain this almost complete connectivity over time across different runs. This rapid increase suggests that nodes efficiently adjust their transmission ranges to form a single-component network, regardless of initial configuration. This consistency across diverse starting conditions highlights the adaptability of the network model in achieving connectivity.

For the mobile adaptive nodes, random movement dynamics introduce further variability; however, the network still reaches stable states, evidenced by the convergence patterns and error bar ranges. This suggests that the adaptive mechanism effectively compensates for node movement, maintaining a fully connected network. However, the slight fluctuations observed indicate periodic adjustments as nodes move in and out of communication range.

Average range and energy usage: The scaled transmission range in Fig. 2a and c increases as the network seeks connectivity but stabilizes once 100% connectivity is achieved. This stability minimizes further range adjustments, conserving energy. Energy usage, derived from the transmission range, in Fig. 2a and c stabilizes after reaching full connectivity, showing the balance between connectivity and energy efficiency. The initial increase in energy usage aligns with the growth phase in connectivity, as nodes expand their range to establish links. As connectivity stabilizes, energy usage also plateaus, demonstrating that nodes no longer need to increase their range, leading to efficient energy usage across different runs. Mobile nodes, challenged by random movement, still maintain low energy use while adapting their range to compensate for movement-induced topology changes.

Link count ratio: The link count ratio Fig. 2a indicates the fraction of active links relative to possible links. A stable ratio reflects efficient use of links, avoiding unnecessary connections even when full connectivity is achieved. It follows trends similar to connectivity and stabilizes at a low value. Once the network achieves full connectivity, further link formation is unnecessary. As the network reaches such state of equilibrium, new connections and disconnections balance dynamically to maintain stable connectivity. This indicates that the network achieves connectivity efficiently, with much fewer links than a fully connected graph, thus conserving energy and reducing complexity.

The mobile network Fig. 2c maintains a similar pattern, demonstrating adaptability with minimal links and without excessive redundancy, regardless of starting conditions. It stabilizes at a level comparable to the static network, though with visible fluctuations reflecting the transient connections formed as nodes move. This fluctuation indicates that while the network achieves a high degree of connectivity, link stability is periodically disrupted and re-established due to mobility.

Coverage: Coverage is crucial as it reflects the spatial reach of the network over the monitored area. Figure 2b and d reveal that both networks eventually can fully cover the domain area once connectivity stabilizes, reflecting efficient spatial adaptation. For mobile nodes, the slight variability due to random movement dynamics reflects occasional gaps in coverage, which are quickly compensated as the network adapts.

Clustering coefficients: Clustering measures the tendency for nodes to form tightly knit groups. The high global clustering in both networks reflects a robust structure with redundancy, which enhances fault tolerance. Local clustering shows that neighboring nodes are also interconnected, adding stability at the local level. Global and local clustering coefficients in Fig. 2b and d indicate network cohesion. These high clustering values demonstrate that the network balances connectivity and redundancy, making it more resilient to disruptions. Mobility slightly reduces clustering in Fig. 2d , as moving nodes periodically alter local neighborhoods.

Average degree: The average degree demonstrated in Fig. 2b and d rises with connectivity but stabilizes at a moderate value, reflecting the network’s optimization in connecting nodes without excessive energy expenditure. Higher degree values would imply more energy usage, while lower values could reduce robustness. By achieving a balanced degree, the network maximizes connectivity while reducing unnecessary links.

In comparing static and mobile networks, both types reach similar stable states, but the mobile network exhibits slightly more variability, as indicated by broader fluctuated error bars. This reflects the challenges due to changing topology introduced by movement, where nodes constantly adjust their transmission ranges to preserve connectivity. This random movement approach with varying velocities provides a realistic stress test for the network’s ability to self-organize and maintain connectivity autonomously. The early rapid adjustments followed by stabilization across all metrics confirm that, even with different initial configurations, the network self-organizes to achieve full robust connectivity with minimal, efficient transmission ranges. Overall, These plots collectively highlight the adaptive algorithm’s effectiveness in achieving a balance between connectivity and energy efficiency, even when faced with random dynamics.

**Fig. 3:**
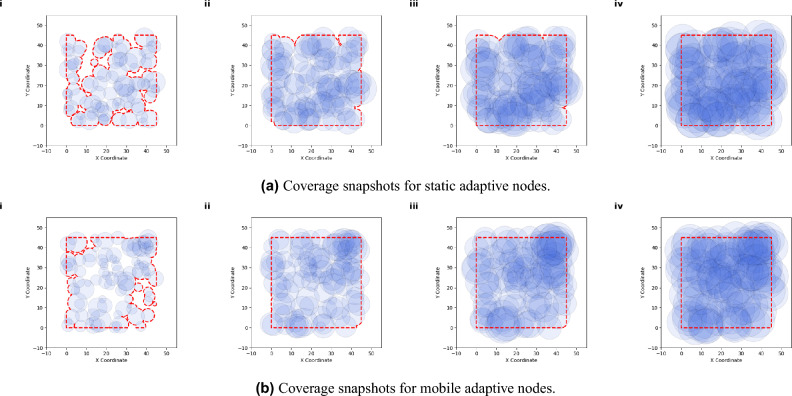
This figure illustrates the evolution of network coverage over time for two types of adaptive networks: static (**a**) and mobile (**b**). The snapshots are taken at steps 0, 200, 500, and 2500, demonstrating how each network adapts its coverage area as nodes adjust their transmission ranges. The blue circles represent the transmission range of individual nodes, while the red dashed outline shows the boundary of the total covered area. In both cases, the networks begin with partial coverage and progressively expand their reach, achieving full coverage.

In this phase of the network”s process, we examine the spatial coverage of adaptive nodes at various stages. Figure 3 visualizes the spatial coverage snapshots of static and mobile networks over time. As shown in Fig. 3a , the nodes initially cover small, isolated areas within the network, as indicated by scattered blue transmission circles within the area. Over time, as nodes adaptively adjust their transmission ranges, the coverage regions expand and increasingly overlap, indicating enhanced connectivity and robustness in the network. The red dashed lines, representing the coverage boundary, reveal the spatial reach of the network over time.

Mobile network Fig. 3b compared to the static network, seems to achieve broader and more consistent coverage in fewer steps. This resilience makes the mobile network suitable for dynamic environments, where maintaining coverage in the face of movement is crucial. Achieving stable boundaries demonstrates that both networks can sustain a high-quality spatial reach. This observation ensures full network coverage besides supporting the overall goals of the study. The achievement of full coverage occurs autonomously, driven by the adaptive mechanisms embedded in the network’s design. This snapshots illustrate that the adaptive algorithm enables both networks to achieve extensive coverage while balancing stability (in the static network) and adaptability (in the mobile network).

**Fig. 4:**
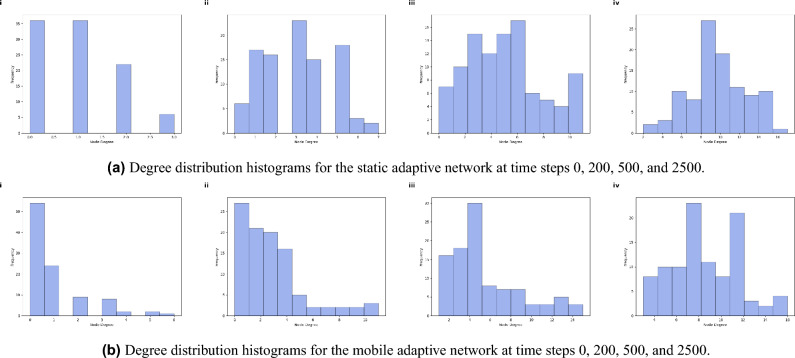
Degree distribution histograms of the adaptive complex network, comparing static and mobile node scenarios at different time steps (0, 200, 500 and 2500). The histograms show the evolution of node connectivity (node degree) as the adaptive algorithm increases network connectivity while maintaining energy efficiency. Subfigure (**a**) represents the static network, while subfigure (**b**) represents the mobile network, illustrating the impact of node mobility on the degree distribution over time.

The histograms in Fig. 4 illustrate the evolution of node degree distribution over time in a complex network of adaptive nodes. These degree distribution histograms provide insights into the adaptive process of the network. At the initial step (i) shown in Fig. 4a , the network begins with a relatively low and uneven degree distribution, reflecting a starting configuration where nodes have yet to optimize their connectivity. As the steps progress (ii-iii), the network undergoes self-organization through adaptive adjustments in each node’s transmission range, leading to changes in the degree distribution. By step 2500 (iv), the degree distribution stabilizes, achieving a broader range of node degrees with a higher average degree than the initial state. This analysis supports the concept of an adaptive mechanism to balance connectivity with potential energy costs.

Mobile networks Fig. 4b exhibit a similar trend but with greater variability due to movement dynamics. However, random movement introduces additional variability, seen in the degree distribution’s less consistent shape across snapshots. This variation indicates the dynamic challenges of maintaining connectivity under mobility. The evolution of these degree distributions aligns well with the goals of maximizing connectivity and robustness. For both static and mobile scenarios, the adaptive algorithm effectively manages connectivity, achieving a adequate degree count without excessive redundancy, supporting efficient energy use. The results provide evidence that the algorithm efficiently handles both scenarios, validating the network’s adaptability and robustness.

**Fig. 5:**
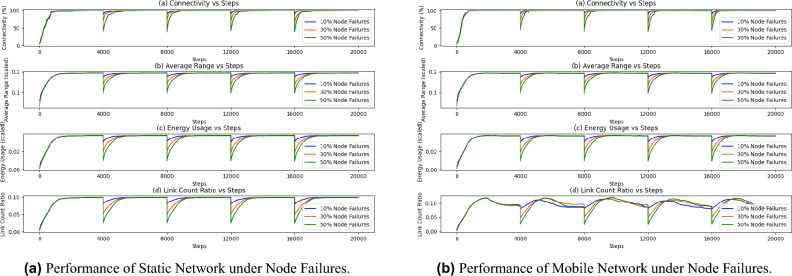
Adaptive network performance under node failures in static and mobile configurations. (**a**) Shows results for a static network with node failures, while (**b**) illustrates a mobile network. Each subfigure presents the evolution of connectivity, average transmission range, energy usage, and link count ratio over simulation steps under scenarios of 10%, 30% and 50% node failures (blue, orange, and green lines, respectively). The adaptive mechanism allows both networks to recover connectivity over time.

To simulate realistic scenarios and assess the network’s resilience, we incorporate node failures as part of the evaluation framework. Node failures serve to model situations in which some nodes become non-functional, either due to hardware issues, battery depletion, or intentional attacks, and thus temporarily lose their ability to communicate within the network. Unlike traditional simulations where failed nodes are permanently removed from the network, our approach includes a recovery mechanism that allows affected nodes to rejoin the network by recalculating and adjusting their transmission range based on the current network conditions. By allowing nodes to reintegrate, we aim better observe the network’s capacity to recover from disruptions and maintain critical connectivity metrics.

Failures are induced in a distributed, random manner: each node is assigned a failure probability and failures are realized independently by local random draws (no centralized controller). A failed node *i* is modeled by setting $r_i(t)=0$ (instant isolation), so it loses all incident links. All nodes predict their transmission range for the next time step at every simulation step; consequently a disabled node may autonomously predict a proper range and gradually rejoin the network. Upon recovery the node follows the standard admission procedure: it becomes a potential neighbor for nodes within its range, and a realized link forms only after bilateral acceptance. Nodes continuously re-evaluate neighbors at each simulation step: when the geometric prerequisite $d_{ij}(t)\le \min (r_i(t),r_j(t))$ no longer holds an link is dropped, and when a new potential contact appears it triggers the bilateral admission check.

Figure 5 examines network performance under failures of 10%, 30%, and 50% of nodes. In both scenarios, displayed in Fig. 5a and b , connectivity initially drops upon failure but recovers as nodes dynamically adjust their ranges. Energy usage temporarily increases during recovery but stabilizes efficiently. Two other metrics also show the similar trends. The analysis demonstrates that the network exhibits a strong capacity for self-healing and restoration of connectivity when subjected to significant node failures (up to 50% of nodes). This self-organizing behavior emphasizes the network’s robustness, as it consistently re-establishes full connectivity even under significant disruptions and adverse conditions. These findings support the utility of the proposed adaptive mechanism in applications requiring reliable and energy-efficient connectivity under dynamic conditions.

**Fig. 6:**
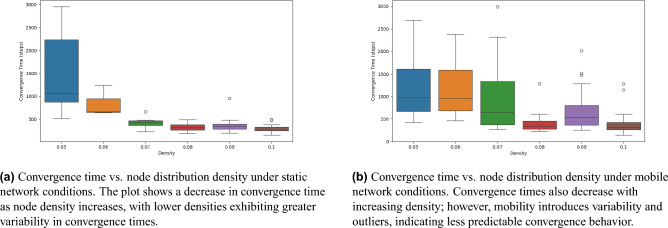
Convergence time versus node distribution density for static and mobile network conditions. Each box plot shows the distribution of convergence times across different density levels, illustrating the impact of node density on network connectivity dynamics under local (**a**) and movement-induced (**b**) conditions.

All the figures and simulations presented so far correspond to networks with a distribution density of 0.05. However, the convergence time required to achieve full connectivity may be influenced by node distribution density in the area. To investigate this potential dependency, Fig. 6 is included to analyze and determine whether such a relationship exists. In the static network, (See Fig. 6a) convergence time decreases remarkably with increasing density, demonstrating that a denser distribution of nodes enables quicker establishment of network-wide connectivity. Lower densities, particularly at 0.05 and 0.06, exhibit a larger spread in convergence times, reflecting greater variability in the network’s ability to converge. This may be due to isolated nodes or smaller clusters that prolong the time required to integrate all nodes into a single connected component. In contrast, at higher densities (0.08 and above), the convergence times become both shorter and more consistent, indicating that increased node density promotes stability and efficiency in connectivity formation.

In the mobile network, Fig. 6b , a similar trend of decreasing convergence time with higher density is observed; however, the results show increased variability across all density levels. At moderate densities (e.g. 0.07), convergence times are widely spread, suggesting that node mobility introduces additional challenges to the convergence process. Movement dynamics can lead to temporary disconnections and reconnections, adding unpredictability to the time required for the network to reach full connectivity. This effect is evident in the larger spread of data points and the presence of outliers across various densities, highlighting that while mobility may offer flexibility, it complicates the process of establishing stable, connected network states. Overall, these findings suggest that while increasing density generally improves convergence times in both static and mobile networks, mobility requires further consideration due to its potential to disrupt network stability, particularly at lower and moderate densities.

**Fig. 7:**
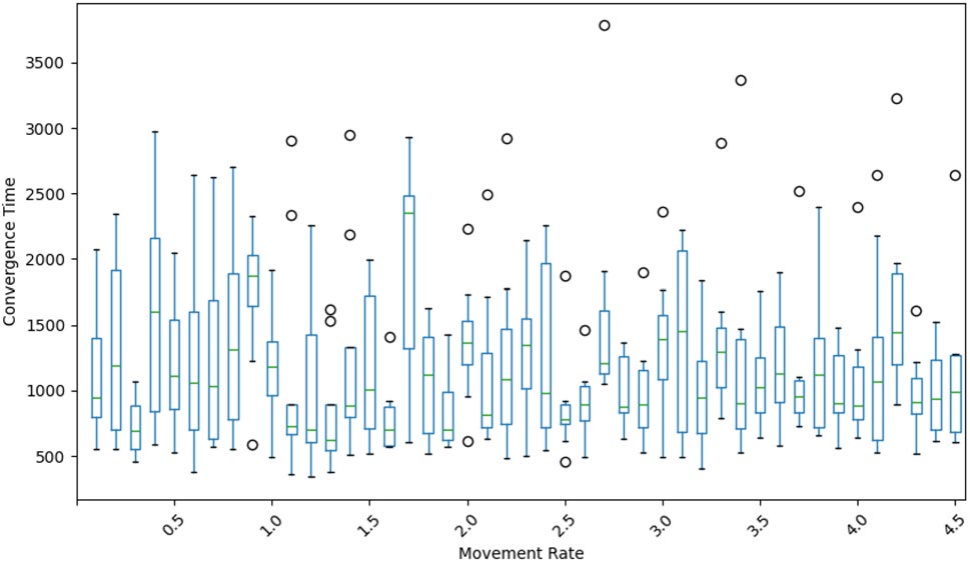
Box plot showing the distribution of convergence times for varying movement rates. Each box represents the range of convergence times observed for a specific movement rate, with outliers depicted as individual points. The movement rate here defines the maximum speed assigned to each node, which follows a normal distribution. This figure illustrates the complex relationship between node speed and convergence efficiency in the network.

The box plot in Fig. 7 highlights the variability and complexity of convergence times across different movement rates in the network. In these simulations, each node is assigned a random velocity, generated using a normal distribution with a defined velocity limit. Here, the movement rate refers to the velocity limit applied to the random velocities of nodes, providing insights into how node mobility influences convergence dynamics. Notably, the convergence time exhibits significant variation at each speed limit, with wide interquartile ranges and numerous outliers. This variability suggests that network convergence is sensitive to node movement, as the continuous changes in node positions influence connectivity and, consequently, the time required to reach full convergence. However, a direct correlation between increased movement rate and faster or slower convergence time is not evident.

The presence of multiple outliers across all movement rates in Fig. 7 further underscores the unpredictability of convergence, as certain configurations or node distributions lead to exceptionally high or low convergence times. These findings suggest that while node mobility impacts network adaptation, achieving optimal convergence likely requires considering additional parameters beyond movement rate alone. While increased mobility introduces challenges, the adaptive algorithm maintains robust convergence across a range of speeds.

**Fig. 8:**
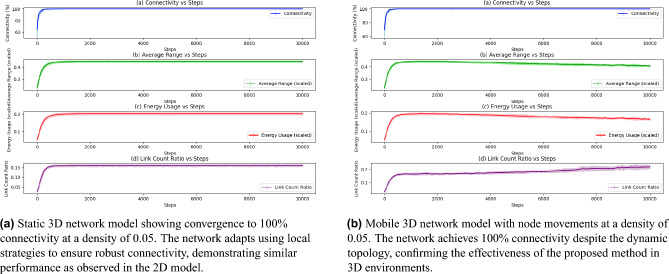
Demonstration of the adaptability and robustness of the proposed method in a 3D network model. The figure consists of two sub-figures: (**a**) a static 3D network with local adaptation, and (**b**) a mobile 3D network with dynamic node movements. Both networks are initialized with the same conditions as the 2D model at a density of 0.05. The results confirm that the proposed method successfully achieves 100% connectivity in 3D network scenarios, validating its generalizability across spatial dimensions.

To demonstrate the generalization capability of our approach, we aim to validate its applicability to three-dimensional (3D) network models in both static and mobile scenarios. The results are depicted in Fig. 8. Figure 8a illustrates that the method successfully achieves 100% connectivity in a static 3D network with local adaptation, mirroring the performance of the 2D model. Similarly, Fig. 8b demonstrates that the network maintains its adaptability in a mobile 3D scenario, overcoming challenges posed by dynamic node movements and topological changes. Stabilized transmission ranges and energy usage reinforce the algorithm’s energy efficiency. The consistent performance across all main metrics highlights the generalizability of the approach without compromising connectivity or performance, even when transitioning from 2D to 3D scenarios. These findings underscore the versatility of the approach and its potential applicability in diverse real-world environments requiring adaptive and resilient 3D network models.

**Fig. 9:**
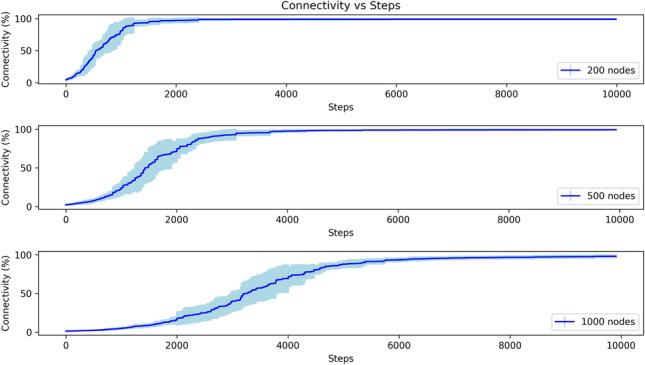
Connectivity versus simulation step for networks of different sizes ($N=200,500,1000$) with static nodes at a distribution density of 0.05. Curves show the ensemble mean of the connectivity metric averaged over 10 different initial node configurations; shaded bands indicate standard deviation across those runs. For all sizes, the qualitative behaviour is preserved. All other model parameters are identical across sizes. The modest increase in apparent convergence time for larger *N* is explained by our time-step convention (A single simulation step is defined as one round in which every node has the opportunity to attempt a change in its transmission range): the per-step probability that any particular node is selected scales as 1/*N*, so the expected number of node updates per simulated step decreases with increasing *N*.

Figure 9 shows the connectivity metric for static networks of size $N=200,500,$ and 1000 to demonstrate performance beyond the 100 node case. The qualitative behaviour observed at $N=100$ is preserved at larger system sizes: connectivity begins near zero, rapidly increases to effectively full connectivity, and thereafter remains in a stable regime with only small local fluctuations. The key quantitative difference is a systematic lengthening of the transient (the time required to reach steady state) as *N* grows. This behaviour is a direct consequence of our time discretization: the per-step probability that any given node is updated is 1/*N*. As a result, the expected number of steps required to effect $\mathcal {O}(1)$ updates per node scales roughly with *N*, which explains the slower apparent convergence for larger networks when plotted against raw simulation steps. Importantly, the long-time (steady-state) connectivity and the small-scale fluctuations around the steady state remain essentially unchanged with *N*, indicating that the connectivity mechanism is intensive and robust to system size for the distributed networks considered here.

We performed a comprehensive literature search for methods operating under the same distributed assumptions and found few that are directly comparable. The baseline Hamiltonian approach^57^ reported connectivity above 95% in similar stylized settings; under the modeling assumptions used here, our method attains connectivity near 100% with noticeably smaller steady-state fluctuations. We explicitly note that these quantitative outcomes depend on the simplified communication and energy model employed, and absolute connectivity values may decrease under richer physical-layer assumptions used in some engineering studies. Nevertheless, the mechanisms and robustness demonstrated by our distributed AI framework address a clear gap in the literature for scalable, self-organizing connectivity maximization.

## Discussion

This study proposed a novel AI-driven self-organizing complex network model for achieving high connectivity, robustness, and energy efficiency in distributed networks. The integration of Hamiltonian-based methodologies with MLP decision-making enabled individual nodes to adapt their transmission power autonomously, optimizing global network performance while responding to local conditions. The results demonstrated the effectiveness of the proposed approach in both static and mobile scenarios, across 2D and 3D environments, confirming its scalability and applicability in diverse contexts. Key findings highlight the network’s ability to achieve 100% connectivity with significant resilience to node failures, dynamic mobility, and varying network densities. The energy usage stabilized at optimal levels, reflecting the efficiency of the adaptive transmission power mechanism. This efficiency not only prolongs the lifetime of individual nodes but also enhances the overall network’s sustainability. Furthermore, the methodology’s ability to balance connectivity and energy consumption underscores its potential for deployment in real-world applications, such as IoT systems, wireless sensor networks, and autonomous communication platforms.

While our results show that AI-driven adaptive nodes can recover high connectivity and improve energy-efficiency across a range of simulated scenarios, several simplifying assumptions limit immediate applicability to real-world deployments:

*Modeling and channel assumptions*: Experiments used a simplified propagation model (free-space path-loss, $n=2$) and did not include fading, shadowing, external interference, or explicit MAC-level contention; packet-level metrics were also omitted. Extending the framework to more realistic channel models (e.g. $\gamma _i r_i^n$ with $n>2$), to include contention and packet-level evaluation, and to adopt cross-layer metrics is an important next step.

*Homogeneity and hardware constraints*: For clarity and reproducibility we simulated homogeneous nodes with identical computation and communication capabilities and continuous transmission ranges. Future work should evaluate heterogeneous device capabilities, discrete transmit-power steps, and empirically measured power curves so that learned policies respect hardware constraints and realistic energy budgets.

*Generalization*: Both MLP$_1$ and MLP$_2$ were trained offline using labels derived from Hamiltonian optimization; as a result, the learned policies may not generalize to environments that depart substantially from the training regimes (different densities, mobility statistics, or channel impairments). A complementary direction is topology-aware modeling: limited-hop GNNs or centrally trained GNNs distilled into compact per-node policies can capture local graph structure while keeping on-device costs low.

*Temporal dynamics*: Our analysis focused on a limited set of mobility and traffic regimes; additional evaluation is needed for bursty mobility models, nonstationary traffic patterns, and abrupt topology changes.

*Physics-guided ML*: A promising complementary direction is to integrate physics-guided machine learning^58^ into the MLPs by incorporating models of signal attenuation, interference, and energy dissipation as inductive biases, constraints, or hybrid physics+ML predictors. This can improve robustness to heterogeneity, energy variation, and noise, ensure physically feasible decisions, and reduce training-data requirements.

*Small hardware testbed experiment*: We plan to implement a modest hardware testbed to (i) compare the Hamiltonian energy proxy with measured device consumption, (ii) measure packet reception rate (PRR), latency, and recovery under realistic MAC contention, and (iii) evaluate robustness under controlled perturbations such as node failures or interference. Lightweight inference models will be deployed on nodes or gateways.

*Trace-based experiments*: In addition to simulations and testbed runs, we will perform trace-replay experiments using public datasets. We will map recorded measurements (e.g. RSSI, PRR, node locations) into our framework and replay the resulting link conditions. This approach will allow us to evaluate the Hamiltonian optimization and distilled policies under realistic, time-varying link behavior. These trace-based results can complement our synthetic simulations and testbed measurements.

By explicitly stating these limitations and outlining concrete mitigation paths, we preserve the contribution of an efficient Hamiltonian-to-ML distillation pipeline for fully distributed inference in complex networks while clarifying the scope of current validation and the roadmap toward practical deployment.

## Methods

The methodology employed in this study is centered on the development and evaluation of a self-organizing complex network model with AI-driven adaptive nodes. The primary objectives are to achieve maximum (100%) connectivity, ensure significant robustness and resilience, and optimize energy usage under varying network conditions. This section elaborates on the dataset generation process, the adaptive learning framework, and the simulation setup used to analyze network performance in both static and mobile scenarios across 2D and 3D environments.

Our network model consists of a distributed complex network of adaptive nodes, each capable of adjusting transmission power to dynamically form or dissolve links with neighboring nodes. The network operates in a fully distributed, ad-hoc manner, with nodes functioning autonomously and without centralized control or predetermined routing protocols. The physical and channel characteristics of nodes in this study are considered within standard operating ranges and are not the focus of analysis. The primary emphasis is placed on the node’s ability to adapt transmission power and connectivity autonomously to optimize network performance.

In our model, nodes are homogeneous in computation and communication capabilities but may adopt heterogeneous, time-varying transmission range $r_i(t)$. A potential contact exists whenever the Euclidean distance $d_{ij}(t)=\Vert x_i(t)-x_j(t)\Vert _2$ satisfies $d_{ij}(t)\le \min \big (r_i(t),r_j(t)\big )$. Network reorganization follows from two mechanisms: autonomous range modification $r_i(t)$ (applies to both static and mobile experiments) and node mobility $x_i(t)$ (applies only to mobile experiments). Nodes continuously re-evaluate environment: when the geometric prerequisite no longer holds an edge is dropped, and when a new potential contact appears it triggers the bilateral admission check.

**Fig. 10:**
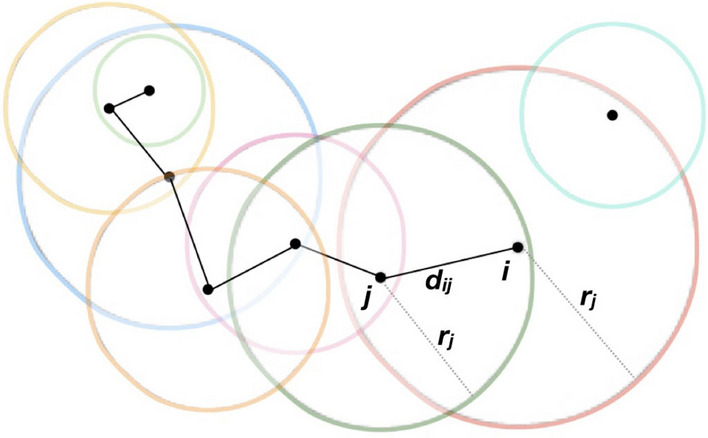
Conceptual illustration of the connection metric. Each node *i* has a transmission range $r_i$ (depicted by circles). A potential link between nodes *i* and *j* depends on their distance $d_{ij}$ relative to their ranges. Links form only when both nodes fall within each other’s range and mutually agree the connection, ensuring adaptive and distributed connectivity^57^.

Potential links are realized only after bilateral admission. When node *i* expands its range so that node *j* becomes a potential neighbor, node *j* evaluates a local acceptance policy and returns $a_j(i)\in \{0,1\}$ . The realized adjacency used in our analyses is therefore

1 $$\begin{aligned} A^{\textrm{real}}_{ij}(t)=\mathbb {I}\!\left( d_{ij}(t)\le \min (r_i(t),r_j(t))\right) \;a_i(j)\;a_j(i), \end{aligned}$$

where $a_j(i)\in {0,1}$ is node *j*’s acceptance of *i*. In our implementation we adopt the convention that the node initiating a range expansion accepts newly discovered neighbors (i.e. $a_i(j)\equiv 1$ for the expanding node *i*), so the realized adjacency reduces to

2 $$\begin{aligned} A^{\textrm{real}}_{ij}(t)=\mathbb {I}\!\big (d_{ij}(t)\le \min (r_i(t),r_j(t))\big )\;a_j(i),\qquad a_j(i)\in \{0,1\}, \end{aligned}$$

where $\mathbb {I}(\cdot )$ is the indicator function. So that a usable link exists if and only if geometry permits mutual detection and both endpoints accept (Fig. 10). So, we adopt the classical Euclidean-distance connection metric as in unit-disk and random geometric graph models^59,60^. However, unlike these deterministic formulations, here link formation is mediated by distributed, independent decision-making.

Nodes can move within a bounded square area, leading to dynamic network topology. Each node is aware only of its local neighborhood (i.e. nodes within its transmission range). Power adjustments impact both the transmission range and energy consumption of nodes, with a trade-off between transmission range and energy efficiency. Network stability is defined by a global state of maximum connectivity, robustness to disruptions, and optimized energy consumption.

To provide each node with a dataset for AI-driven decision-making, we first employed a Hamiltonian-based optimization framework from our previous work to model an optimized network state. In this approach, the Hamiltonian function (*H*) is designed to minimize energy usage while maximizing connectivity and robustness across the network. Each node decides adjustments to its transmission power based on this function, with the goal of steering the network toward a stable state that meets the connectivity, robustness, and energy efficiency conditions.

In classical mechanics, the Hamiltonian provides a scalar measure of system energy and its dynamics. As an energy function whose minimization determines steady configurations, we take this viewpoint and treat the network Hamiltonian as a cost to be minimized. We adapt this idea by defining a Hamiltonian for the network that captures the trade-off between structural and resource constraints. In our formulation^57^ the Hamiltonian for node *i* reads:

3 $$\begin{aligned} H_i \;=\; \alpha _i k_i^2 + \beta _i k_i^3 + \gamma _i r_i^2 + \lambda _i \sum _{j\ne i}\frac{A_{ij}}{d_{ij}}. \end{aligned}$$

Here, $k_i$ denotes the degree of node *i*, $r_i$ its transmission range, $A_{ij}$ the adjacency matrix element (1 if connected, 0 otherwise), and $d_{ij}$ the Euclidean distance between nodes *i* and *j*, and $\alpha _i,\beta _i,\gamma _i,\lambda _i$ are tunable weights. The Hamiltonian of the network is equal to the sum of the Hamiltonians of the individual nodes.

The terms separate into connectivity and energy contributions. Connectivity comprises $\alpha _i k_i^2$, which rewards hub formation^61^; $\beta _i k_i^3$, penalizes excessive centralization while promoting three-node connections^61^; and $\sum _{i\ne j}\lambda _i A_{ij}/d_{ij}$, which incentivize long-distance links to prevent fragmentation. The energy contribution $\gamma _i r_i^2$ models transmission cost via the Friis relation^62^ and penalizes large transmission ranges to ensure energy efficiency. Their interplay balances connectivity and energy, yielding sustainable and globally connected networks. We minimize *H* using a Monte Carlo approach, and declare convergence to a steady state once ensemble observables, such as overall connectivity, become stationary.

In Eq. (3) the term $\gamma _i r_i^2$ serves as a penalty on transmission range and represents the transmission-power cost under a free-space path-loss assumption (path-loss exponent $n=2$). Concretely, the Hamiltonian term captures the trade-off between connectivity and transmit cost: larger ranges improve connectivity but increase the $\gamma _i r_i^2$ penalty, encouraging energy-aware solutions. In the experiments reported here, the same quantity is also used as the proxy for per-node energy when computing evaluation metrics; therefore the numerical energy values should be interpreted as relative costs under the chosen baseline model rather than absolute measures of real device energy consumption.

The following simplifying assumptions were adopted to study the general behavior of distributed range adaptation: (i) free-space propagation with $n=2$ (no fading or multipath), (ii) no explicit model of external interference or network-level contention, and (iii) no hardware-specific maximum transmit-power cap (transmission ranges are bounded implicitly by the simulation domain). These choices were deliberate to focus on the algorithmic behavior of the Hamiltonian framework in complex, distributed topologies. The Hamiltonian formulation is modular: the $\gamma _i r_i^2$ term can be replaced by a more realistic cost model (for example $\propto r_i^n$ with $n>2$, a log-normal shadowing term, or an empirical power-consumption curve that includes circuit overhead and discrete power levels). In such cases, new datasets can be generated by re-running the Hamiltonian optimization under the modified cost model, while the overall MLP-based training and deployment pipeline remains applicable. Minor adjustments to model capacity may be introduced if required by the complexity of the new mapping.

The network should maintain near-complete connectivity, with each node reachable from any other through a series of links. The network should sustain connectivity even in the presence of local failures or node removals. Nodes should operate at minimal energy expenditure, achieved through careful power control. Using this Hamiltonian approach, we generated a dataset comprising optimal transmission power values for various network states. This dataset includes local network topology information, energy consumption metrics, and power adjustments required to achieve optimized network conditions. The Hamiltonian formulation is designed to minimize energy consumption while maximizing connectivity, providing an optimal baseline for training the AI model.

**Fig. 11:**
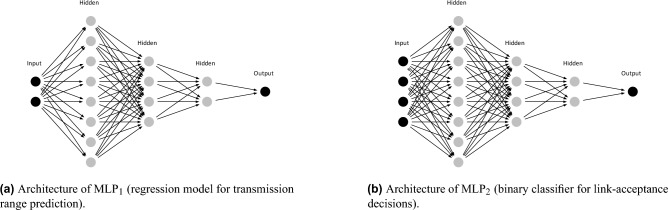
Neural network architectures used in this study.

Building on the dataset generated from the Hamiltonian framework, we designed two MLPs that are deployed identically on every node to enable real-time, adaptive transmission-control and link-formation decisions. We used two lightweight multilayer perceptrons, MLP$_1$ and MLP$_2$. Each model has an input layer, three hidden layers (their sizes were selected based on validation results), and an output layer; both are fully connected networks with ReLU activations in the hidden layers, dropout of 0.1, and batch normalization after each dense block. Training and evaluation were carried out in TensorFlow/Keras (v2.13.1). The schematic diagrams of the MLP architectures are shown in Fig. 11. MLP$_1$ is a regression model that maps local features, including the node degree and the node’s current transmission range to a candidate transmission range. MLP$_1$ was trained with the Adam optimizer (initial learning rate $1\times 10^{-3}$) to minimize the mean absolute error (MAE)

4 $$\begin{aligned} \mathcal {L}_{\text {MLP}_1}(\theta ) \;=\; \frac{1}{N}\sum _{i=1}^{N}\bigl |f_{\theta }(x_i)-y_i\bigr |, \end{aligned}$$

where $x_i\in \mathbb {R}^2$ denotes the input features for sample $i$, $y_i\in \mathbb {R}$ is the true next-range label, and $f_{\theta }(\cdot )$ is the network prediction.

MLP$_2$ is a binary classifier that maps some local features, including the node’s current transmission range, distance to desired node, node degree (local connectivity), and *T* (a metric assigned to each node representing its freedom), to a link-acceptance probability. It was trained with the Adam optimizer (initial learning rate $1\times 10^{-3}$) using binary cross-entropy (BCE) loss,

5 $$\begin{aligned} \mathcal {L}_{\text {MLP}_2}(\theta ) \;=\; -\frac{1}{M}\sum _{j=1}^{M}\bigl [t_j\log p_j + (1-t_j)\log (1-p_j)\bigr ], \qquad p_j \;=\; \sigma \bigl (g_{\theta }(x_j)\bigr ), \end{aligned}$$

where $x_j\in \mathbb {R}^4$ is the input to the classifier for sample $j$, $t_j\in \{0,1\}$ is the binary label, $g_{\theta }(\cdot )$ is the classifier logit, and $\sigma (\cdot )$ is the sigmoid function. During deployment MLP$_2$ outputs were thresholded at $0.5$ for accept/reject decisions, i.e. $\text {accept}\Leftrightarrow p_j\ge 0.5$. The reported evaluation metric was binary accuracy,

6 $$\begin{aligned} \text {Accuracy} \;=\; \frac{1}{M}\sum _{j=1}^{M}\textbf{1}\{\textbf{1}\{p_j\ge 0.5\}=t_j\}. \end{aligned}$$

Both MLPs were trained on a dataset generated by the Hamiltonian-based framework. The dataset sizes differed between the two models: MLP$_1$ used $\approx 7{,}500$ samples after preprocessing, since only range-changing events contribute useful labels, while MLP$_2$ used $\approx 232{,}000$ samples owing to the large number of potential link evaluations generated per simulation. To prevent overfitting the dataset was split into 70% training and 30% validation. Hyperparameters were tuned by random search; the final settings are summarized in Table 1.

**Fig. 12:**
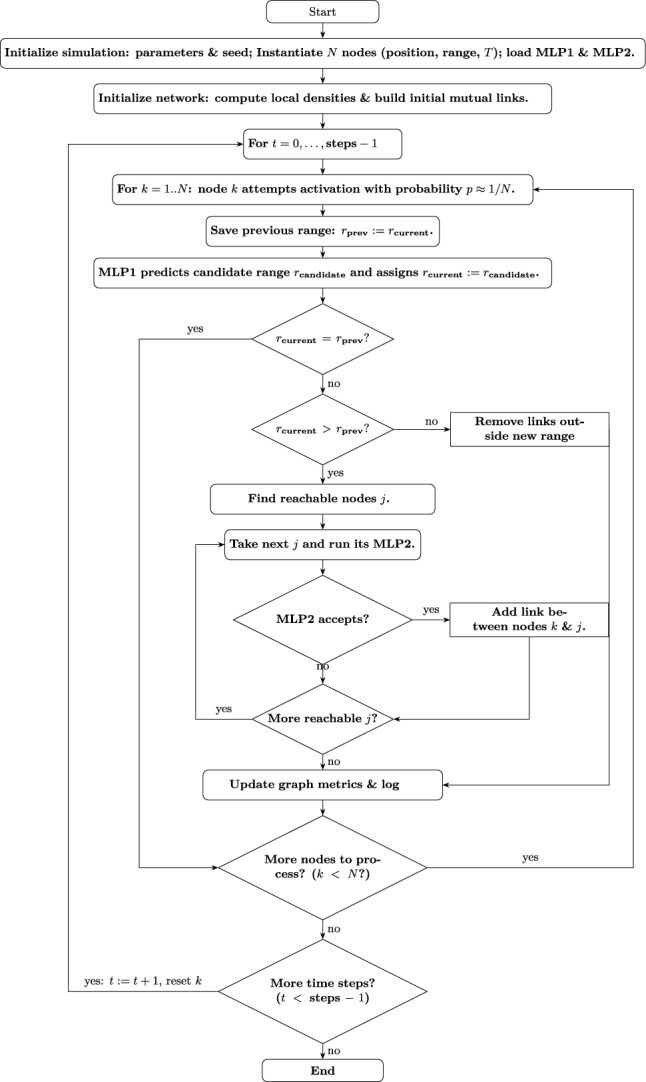
Flowchart of the proposed algorithm for static distributed networks with AI-driven adaptive nodes. Since nodes are static, local density distributions are computed once at initialization and used throughout the simulation. For networks with mobile nodes, densities should be updated at appropriate intervals. Each node independently decides whether to activate at a given time step, and its MLP1 predicts a candidate transmission range and assigns it as the current range. If this range exceeds the previous one, the node may attempt to connect with reachable nodes; these newly reachable nodes independently run their MLP2 to decide whether to accept the new link, considering potential mutual range constraints. Links are disconnected when the current range is smaller than the previous range. Graph metrics are updated and logged after all node decisions in each time step.

So, These models serve as the decision-making core of each adaptive node. By learning the mapping from local topology and resource settings to actions, these per-node policies enable fully distributed operation with minimal computation and communication overhead. The algorithmic pipeline is summarized in the flowchart shown in Fig. 12. Through repeated application of this process, this distributed AI-driven approach allows nodes to self-organize and adapt dynamically to varying environmental and network conditions, demonstrating emergent global behaviors such as high connectivity and resilience against disruptions.

**Table 1:** Summary of MLP architectures and training hyperparameters.

Parameter/item	Exact value/note
A. General settings	
Framework	TensorFlow/Keras (TensorFlow 2.13.1)
Input features (MLP$_1$)	degree, current range
Input features (MLP$_2$)	*T*, current range, degree, distance to candidate peer
Preprocessing	Rows with no range change removed (MLP$_1$); first 10% rows trimmed to remove initialization transients (MLP$_2$)
Train / validation split	70% / 30% (pandas sample(frac=0.7, random_state=0))
B. MLP$_1$ (regression) - architecture & training	
Architecture (layers)	Input (2) $\rightarrow$ Dense(8, ReLU) $\rightarrow$ Dropout(0.1) $\rightarrow$ BatchNorm $\rightarrow$ Dense(4, ReLU) $\rightarrow$ Dropout(0.1) $\rightarrow$ BatchNorm $\rightarrow$ Dense(2, ReLU) $\rightarrow$ Dropout(0.1) $\rightarrow$ BatchNorm $\rightarrow$ Dense(1, linear)
Output	Scalar next-range prediction (linear)
Loss	Mean absolute error (MAE)
Optimizer	Adam (initial LR $=1\times 10^{-3}$; Keras defaults for other Adam params)
Batch size	256
Max epochs	200 (no early stopping)
Dropout	0.1 (after each dense block)
Batch normalization	Applied after each dropout block
Training samples	$\approx 7{,}500$
Best validation metric	MAE = 1.5903 (epoch 181)
C. MLP$_2$ (binary classifier) – architecture & training	
Architecture (layers)	Input (4) $\rightarrow$ Dense(8, ReLU) $\rightarrow$ Dropout(0.1) $\rightarrow$ BatchNorm $\rightarrow$ Dense(4, ReLU) $\rightarrow$ Dropout(0.1) $\rightarrow$ BatchNorm $\rightarrow$ Dense(2, ReLU) $\rightarrow$ Dropout(0.1) $\rightarrow$ BatchNorm $\rightarrow$ Dense(1, sigmoid)
Output	Scalar probability (sigmoid); thresholded at 0.5 for accept/reject
Loss	Binary cross-entropy
Metrics	Binary accuracy (reported)
Optimizer	Adam (initial LR $=1\times 10^{-3}$; Keras defaults)
Batch size	1024
Max epochs	20 (no early stopping)
Dropout	0.1
Batch normalization	Applied after each dropout block
Training samples	$\approx 232{,}000$
Best validation loss (binary cross-entropy)	0.0628 (epoch 19)
Validation binary accuracy	0.9858 (98.58%)

We conducted simulations with multiple random seeds to ensure statistical reliability: ten distinct seed values were used across runs. The experiments were implemented in Python with standard scientific libraries (NumPy, iGraph, Matplotlib, Pandas) and neural models loaded from saved Keras model files. Each simulation used 100 nodes by default, and we varied the global average node density across the range 0.01 to 0.10; for a given density the domain size was chosen so that the number of nodes matched the specified density (for two-dimensional runs the deployment area was square, with side length equal to the rounded square root of nodes divided by density; for three-dimensional runs the domain was cubic with side length equal to the rounded cube root of nodes divided by density). While 100 nodes was the default configuration for the experiments reported in the main plots, we also repeated selected experiments with 200, 500 and 1000 nodes to verify that our findings scale consistently.

Nodes were placed uniformly at random inside the domain; each node’s initial transmission range was set around a baseline mean of 3 units with a small Gaussian perturbation, and positions (and heights for three-dimensional runs) were sampled from a uniform distribution. Each node was also assigned an internal parameter *T* drawn as a multiple of 100 between 0 and 900, and in mobile scenarios velocities were drawn uniformly from the interval $\pm 0.5$ (static scenarios used zero velocity). Time advanced in discrete steps with a time increment of one unit per step; positions were updated by adding velocity times the time step and reflections were used at domain boundaries.

Local node density for each node was estimated by counting neighbors within radius *R*, 2*R* and 3*R*, converting those counts to densities by dividing by the relative areas (using $S = \pi \cdot \texttt {range}^2$), and averaging those three values to two decimal places to obtain local density. Connectivity used a symmetric neighbor rule. Simulations typically ran for 10,000 or 20,000 steps, and at each step nodes were considered for update with probability equal to the reciprocal of the total number of nodes (so on average one node was updated per step); after node updates the graph and relevant metrics were recomputed.

To assess the effectiveness of the AI-driven self-organizing network, we evaluate network performance based on the following metrics:

- *Connectivity ratio*: The percentage of nodes connected within a single giant component of the network, reflecting the network’s overall connectedness.
- *Robustness*: Measured by the network’s resilience to node failures, defined as the ability to maintain connectivity when a percentage of nodes or links are removed.
- *Energy consumption*: The average energy usage per node, which captures the network’s efficiency in power management. The energy consumed of node devices considered inversely related to the square of the distance as $power \propto \frac{1}{r^2}$, based on the *Friis transmission equation*^63^.
- *Link count ratio*: A metric used to evaluate the connectivity of a network by comparing the actual numbers of links in the network to the total possible number of links. It provides a measure of how densely the nodes in a network are connected.
- *Adaptability*: The network’s ability to reorganize and maintain optimal performance when faced with local disturbances, such as node mobility or varying density.

The proposed model was evaluated through extensive simulations conducted in Python. The simulations were designed to compare network performance across static and mobile nodes in both 2D and 3D network environments. Key parameters included:

- *Node distribution densities*: Simulations were conducted for varying densities to assess the scalability of the model.
- *Mobility dynamics*: Random movement patterns were introduced for mobile nodes, with speeds sampled from a normal distribution.
- *Node failures*: Scenarios were simulated with up to 50% node failures to test the resilience of the network.

Each simulation tracks critical performance metrics. For mobile networks, the model is expected to demonstrate adaptability by responding to dynamic topology changes, with nodes recalibrating their transmission power based on real-time local conditions. The outcomes confirm that the proposed method achieves stable and maximized connectivity while maintaining low energy usage.

## Data Availability

The datasets used in this study are available at https://github.com/Azra-Syd/Distributed-Complex-Networks-with-AI-Driven-Adaptive-Nodes/tree/main.
